# Urdu Receptive Language Scale (URLS): Modification & development of protocol for administration in Urdu

**DOI:** 10.12669/pjms.40.5.7471

**Published:** 2024

**Authors:** Ghazal Awais Butt, Nazia Mumtaz, Ghulam Saqulain

**Affiliations:** 1 Ghazal Awais Butt, PhD Scholar Speech & Language Pathologist, Department of Rehab and Allied Health Sciences, Riphah International University, Lahore, Pakistan; 2 Nazia Mumtaz, PhD (Rehabilitation Sciences) Head of Department & Professor, Department of Speech Language Pathology, Faculty of Rehab and Allied Health Sciences, Riphah International University, Lahore, Pakistan; 3 Ghulam Saqulain, FCPS (Otorhinolaryngology) Head of Department & Professor, Department of Otorhinolaryngology, Capital Hospital PGMI, Islamabad, Pakistan

**Keywords:** Child, Language, Pretesting, URLS, Modification

## Abstract

**Objective:**

To conduct pretesting of Urdu Receptive Language Scale (URLS) for scale modification and development of protocol for administration on children.

**Methods:**

This exploratory research was conducted from December 2021 to June 2022 in Lahore, Pakistan. Objective was achieved by pretesting in three steps including literature review, expert review and pilot testing on infants, toddlers and children aged 0-6 years utilizing purposive sampling technique. For expert review five experts’ speech language pathologists with minimum five years of experience and for pilot study 48 normal developing children age range birth to six years were recruited. The analysis was done using content validity ratio, content validity index and Cronbach alpha. Protocol for administration of test items and stimulus with minimum three correct responses among four children’s responses were finalized.

**Results:**

Of the 59 items, 57 with CVR = 0.99 were retained. Item number 3 (age 6-11 months) and item 5 (age 3.6-3.11 years) were eliminated as CVR was <0.99. CVI was one for retained items indicating acceptable validity. Cronbach’s alpha was 0.95 indicating high level of internal consistency for the scale. Task groups of protocol of administration directions included: Selection of scale items, testing environment, seating arrangements, rapport building and involving the caregiver, handling the scale material and scoring the task responses.

**Conclusion:**

The modified 57 items has high validity and internal consistency with suitable protocol of administration. It is linguistically appropriate for the application on larger scale with children of different cultural backgrounds in Pakistan.

## INTRODUCTION

Language development starts early in the life of human beings, necessitating consideration of the language development milestones of children to timely identify any deviation or delay in language development in the early years of life.^1^ Receptive and expressive language development determines the expressive effect size at different ages.^2^ Receptive language means understanding of language and any information presented in various ways such as words and sounds, gestures and movement and symbols & signs. It is worth noting that children achieve elements of receptive language at a faster rate than expressive language.^3^

The ability to communicate using language or the process of development of language consists of three steps. In the first step the child needs exposure to words or sounds repeatedly to become familiar with these sounds or words. In the second step the child starts associating these words or sounds with the object they represent and in the final step the child starts to make use of these words in the environment.^4^ Speech language pathologists (SLP) need to draw conclusions about individuals’ communicative abilities and hence it is essential to make assessment to deal with various kinds of speech, language, communications and swallowing problems.^5^ SLPs use informal as well as formal methods of assessment and also a combination of both to evaluate communicational abilities, however the paths to that may vary.

There are several methods and approaches that include norm-referenced tests, criterion-referenced tests and authentic assessment approaches. Urdu, being the national language of Pakistan is very different from English language in morphology and syntactic structure. There are more than 11 million persons whose mother tongue is Urdu and at the same time it is used as a second language by more than 105 million people.^6^ Preschool screening for developmental difficulties is increasingly becoming part of routine health services provision yet the scope and validity of tools being used within these screening assessments is variable. Sim F. at al in 2019 reviewed numerous tests available for language assessments across different countries and reported the predictive validity of preschool tools used for screening of behavior difficulties and language in community settings.^7^

A local study conducted in the year 2021 on the common speech and language screening/assessment or diagnostic tools used by speech and language pathologist in Pakistan reported that Pakistani SLPs are not using linguistically appropriate assessment tools for speech & language disorders, hence there is a need to develop appropriate assessment tools for Urdu speaking Pakistani population.^8^ Content validity and internal consistency measurements of tools is essential and hence designing of different assessment scales should be found with validity being a crucial factor in applying or selecting an instrument. It is often determined in three forms i.e. construct, content and criterion-related validity.^9^ Sufficient literatures is available on various questionnaire development and validation studies in fields of Allied Health Professionals in Pakistan.^10^-^12^ but limited literature for receptive and expressive language assessments tools that can help in diagnosis of language disorders.

Literature available on the development of language demonstrates that language screening & assessment is a crucial source of information regarding the intervention techniques used for children with any language disorder or impairment.^13^ Many assessment tools are available for English language that are valid and reliable according to English norms. In Pakistan first Urdu language tool to assess receptive language was developed in 2016 named as Urdu Receptive Language Scale (URLS) for children from birth to six years of age with scale items divided into twelve sections with six-month age difference between each age range and with each age group having a distinct number of items. These test items focused on listening, attention, understanding questions, command following, morphological patterns and deriving simple conclusions.^14^

Other tests available in Urdu language including Pakistani tests like Test of Articulation and Phonology (TAPU), Action Picture Test (APIT), Aphasia Naming Test in Urdu, Urdu Speech Perception Test (USPT) and (U-PPVT-4) an Urdu version of Peabody picture vocabulary test but there is a lack of standardized measures for language assessments in Pakistan^15^ with a huge population of children less than five years of age (14.80%).^16^ This literature gap led the authors to work further to conduct pretesting of Urdu Receptive Language Scale (URLS) for modification & development of protocol for administration to make it applicable in the recent era and embarking further on norm referencing process. The study is important since it will help provide a valid, reliable, inexpensive and efficient screening or assessment tool for Pakistani Urdu language speaking children.

## METHODS

This Exploratory study was conducted over a period of six months from 14^th^ December, 2021 to 15^th^ June, 2022 with a sample of five experts and 48 children. Five Speech and language pathologists of both genders with a minimum of five years’ post-graduate experience were recruited using purposive sampling for expert opinion from Lahore and Islamabad, while n=48 children of both genders aged birth to six years with normal language development and Urdu as their native language, were included using convenience sampling from two day care centers including Riphah daycare center and Alifya early years (preschool & daycare) and one mainstream school i.e., the Spirit School Lahore. Sample included four children for each age group. Experts working on administrative positions and children diagnosed with any speech and language disorder, behavioral problems including any inappropriate social behavior like hitting, spitting, harmful behavior and psychological problems were excluded from the study.

### Ethical Approval:

Study was carried out following ethical approval of Institutional Research Board of Riphah International University vide Reference no. REC/RCR S/16/3001. Written consent was obtained from experts. Parents’ informed consent was also obtained and voluntary participation and decision of withdrawal from research was kept open for them.

**Fig.1:**
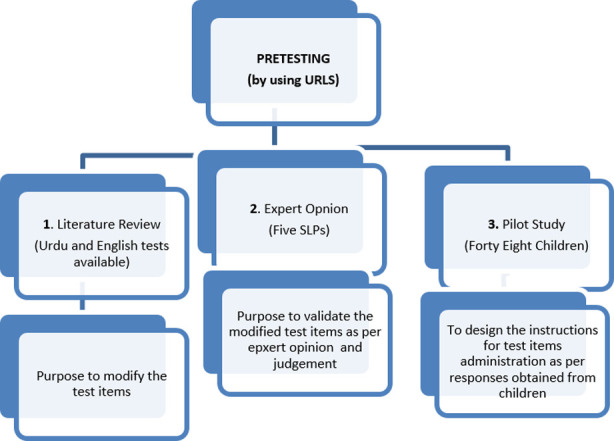
Methodology.

Demographic information was collected from experts and parents of children included in study. It was assured that no psychological or physical harm was caused to participants during data collection. Demographic sheet and URLS test items^11^ were used for data collection from experts, while demographic sheet, URLS test items, manipulative objects and colored pictures were used for data collection from children. Data collection involved pretesting of URLS including following steps (Fig.1):-

### Literature Review:

The age range for children was 0-6 years for Urdu Receptive Language Scale (URLS). These age ranges were modified as per literature and divided into 12 age levels; 0-5 months, 6-11 months, 1-1.5 years, 1.6-1.11 years, 2-2.5 years, 2.6-2.11 years, 3-3.5 years, 3.6-3.11 years, 4-4.5 years, 4.6-4.11 years, 5-5.5 years and 5.6-5.11 years. Domains for receptive language assessment were finalized as focusing on attention, play behavior, understanding of gestures, morphology, semantics and syntax. The test items were also reordered as per age range modification and literature review.

### Expert Review:

For modified test items validation, content validity was assessed by calculating Content Validity Ratio and Content Validity index. For CVR experts were required to make judgment on the four-point scale rating i.e 1. Essential, 2. May be essential, 3. Useful but not essential, 4. Unnecessary. On the basis of CVR values calculated by using formula CVR = (ne-N/2)/N/2 and items with less than 0.99 value were eliminated as per Lawshe table method for five expert ratings. CVI was calculated for retained items by using the formula CVI = ΣCVR/Ncvr along with this internal consistency of test items also being checked by measuring Cronbach alpha and the items were finalized.

### Pilot study:

It was conducted on 48 children with normal language development divided in twelve age brackets, so there were four children selected in each age group. As test items were different for all age groups so each child was presented with the appropriate task and their responses were recorded accordingly. A minimum three out of four correct responses for test items directions were finalized in the form of written instructions. For gathering pilot study responses for test items children were assessed in multiple sittings as per their comfort level. Suitable Stimulus for each test item was presented either in picture form or in the form of manipulative object or verbal instruction. After noting correct responses minimum three out of four, the most suitable stimulus was selected for each test item and recorded in the manual in the form of written instruction/ protocol for administration. Finally, after pilot study test items along with instruction manual were finalized so that it can become applicable on larger sample of children.

### Statistical Analysis:

The SPSS version 21 was used for data analysis. Descriptive statistics were utilized and frequencies, percentage of demographic data and CVI, CVR and internal consistency was confirmed by Cronbach’s alpha measured for test item analysis. Protocol for administration was developed and directions for test item and stimulus with minimum three correct responses among four children’s responses were finalized during the pilot testing.

RESULTS

In the current study experts included a total of 05 Speech Language Pathologists (SLPs) of which 03 had MS SLP qualification with experience of more than 10 years and another two SLPs were PhDs with one having experience of between 6-10 and the other more than 10 years. Sample for pilot study included 12 age groups for 48 children with four children in each group with equal gender distribution. There were total 57 test items and each age range had distinct test item distribution (Table-I).

**Table-I:** Age wise Frequency distribution of Test Items (n=57).

Age range	Test items
0-5months	5
6-11 months	6
1-1.5years	5
1.6-1.11years	5
2-2.5years	6
2.6-2.11years	5
3-3.5years	5
3.6-3.11years	4
4-4.5years	4
4.6-4.11years	4
5-5.5years	4
5.6-6years	4

Total items	57

CVR was calculated for all items and item number three for age group 6-11 month and item number five for 3.6 to 3.11 years were eliminated due to CVR of 0.2 and 0.6 respectively (Table-II). These eliminated items had content validity ratio lower than 0.99 (according to the expert numbers in our study that was five, numerical values of the Lawshe table was 0.99) or those which combined to remaining items based on the opinion of content experts through editing of item. CVI for the retained items was calculated as mentioned below.

**Table-II:** Age wise distribution of Content Validity Ratio (CVR) of Test Items (n=57).

Age Group	Items	Ne	CVR	Interpretation	Age Group	Items	Ne	CVR	Interpretation
0-5 Months	1	5	0.99	Retained	3-3.5 Years	1	5	0.99	Retained
	2	5	0.99	Retained		2	5	0.99	Retained
	3	5	0.99	Retained		3	5	0.99	Retained
	4	5	0.99	Retained		4	5	0.99	Retained
	5	5	0.99	Retained		5	5	0.99	Retained
6-11 Months	1	5	0.99	Retained	3.6-3.11 Years	1	5	0.99	Retained
	2	5	0.99	Retained		2	5	0.99	Retained
	3	4	0.2	Eliminated		3	5	0.99	Retained
	4	5	0.99	Retained		4	5	0.99	Retained
	5	5	0.99	Retained		5	5	0.6	Eliminated
	6	5	0.99	Retained	4-4.5 Years	1	5	0.99	Retained
						2	5	0.99	Retained
1-1.5 Years	1	5	0.99	Retained		3	5	0.99	Retained
	2	5	0.99	Retained		4	5	0.99	Retained
	3	5	0.99	Retained	4.6-4.11 Years	1	5	0.99	Retained
	4	5	0.99	Retained		2	5	0.99	Retained
	5	5	0.99	Retained		3	5	0.99	Retained
1.6-1.11 Years	1	5	0.99	Retained		4	5	0.99	Retained
	2	5	0.99	Retained	5-5.5 Years	1	5	0.99	Retained
	3	5	0.99	Retained		2	5	0.99	Retained
	4	5	0.99	Retained		3	5	0.99	Retained
	5	5	0.99	Retained		4	5	0.99	Retained
2-2.5 years	1	5	0.99	Retained	5.6-6 Years	1	5	0.99	Retained
	2	5	0.99	Retained		2	5	0.99	Retained
	3	5	0.99	Retained		3	5	0.99	Retained
	4	5	0.99	Retained		4	5	0.99	Retained
	5	5	0.99	Retained					
	6	5	0.99	Retained					
2.6-2.11	1	5	0.99	Retained					
	2	5	0.99	Retained					
	3	5	0.99	Retained					
	4	5	0.99	Retained					
	5	5	0.99	Retained					

The item in age group 0-6months/ 
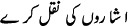
 / was eliminated due to low CVR as few experts suggested it is not directly related to receptive language. Another eliminated item was / 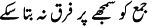 from the age group 3.6-3.11 years as expert believed that this item should be added where the required concept will be achieved and not necessary to be added here. Modifications that were made as per expert judgment includes changing age range in months to years format, mention statements like (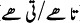) to write test items where required and suggested to use clinical words for few terms like use /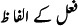/ for/ 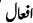 and mention exact required task statement instead of writing /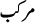/ and /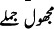 words. CVI for scale was one for retained items that showed the acceptable validity for the scale. It was calculated by using the following formula:


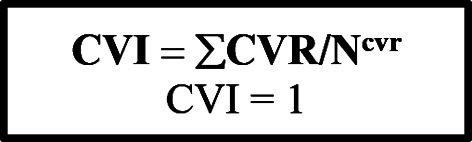


The value of Cronbach’s alpha is 0.95 and it indicates a high level of internal consistency for the scale. After pilot study modified version of URLS is available along with manual for written instructions and stimulus in the form of pictures and manipulative objects for test items administration on children age 0-6 years along with protocol of administration directions with task groups and subgroups as depicted in (Table-III).

**Table-III:** Protocol of Administration / Directions.

Task Group	Task Subgroup
Selection of scale items	Select the test items of a specific age group according to the age of the targeted child.
	Instruction should be read carefully before application.
	All tasks of a specific age range should be applied.
	Tasks should be applied as written in the form of instruction along with the suggested pictures or manipulative objects.
Testing environment	Quiet, comfortable room with plenty of light is desirable.
	Room or testing area should be distraction free i.e. any extra toys or pictures
Seating arrangements	Infants or very young children can sit in mother’s lap, on floor or near to the caregiver.
	For older children table and chair can be used as per the comfort of the child
Rapport building and involving the caregiver	Infants or young children must be given some play time with extra material other than the test material to make them comfortable to response.
	Caregivers or parents can be involved to present the task to infants or young children without giving any hint or cue.
Handling the scale materials	Strict compliance required for using the assigned stimulus either in the form of pictures or manipulative objects.
	Only required material should be in front of the child, keep rest in a box.
	Carefully using or switching the material for presenting different tasks to avoid distractions.
Scoring the task responses	Correct responses as defined in the instruction should be score ‘1’ and score ‘0’ for no or wrong response.
	Count the correct responses for each task to calculate the standard scores and percentile ranks.

## DISCUSSION

The speech, voice, language, feeding and swallowing disorders are quite prevalent with an inflated prevalence among children in the ages of three to six years (11%), in comparison to children with age of 7 to 10 years (9.3%) and children from age 11 to 17 years (4.9%).^17^ Hence, the timely identification of all these disorders is vital for children’s scrutiny^15^, the routine practice of SLPs working with children is to perform accurate diagnostic evaluations. The appropriate selection of tests to be used as part of a diagnostic evaluation can be critical for providing the best clinical services.^18^ Therefore, the current study targeted to modify the items of URLS and develop protocol for administration for the age group of 0-6 year’s children and the modified version of URLS was developed with instructions to apply these items along with stimulus pictures or objects finalized after conducting pilot study. Test items were divided into twelve age brackets with all items related to receptive language domains and used for assessing receptive language of children with language disorders. Validity and reliability results for modified URLS showed high level of scale validity and internal consistency of test items compared to older version.^14^

All designed instructions are mentioned in instruction manual along with description of recorded responses while applying the URLS on children. According to literature review and expert panel suggestions months were converted to year’s format, sentence written expression for test items was changed from subjunctive mood to present participle tense and clinical terms were introduced for a few pure Urdu words used in the test items statements. Previous URLS^14^ is modified in current study to maintain the evidence-based practice for Pakistani SLPs and to encourage them to use recent and best evidence available consistent with studies noted in a systematic review by Denman D et al.^19^ indicating that improvements were the need of the hour. Current study is significant since it has added modified assessment tool to fill the gap of such assessment tools in Urdu language. The tool is useful in diagnosis of receptive language disorders in children and thus it will contribute to promotion of intervention in children. At present there is no other validated tool available for this purpose in Urdu Language.

According to Betz SK et al. the psychometric features of assessment tools do not seem to govern frequency of tool use and evidence-based practice is the need of the hour.^20^ Though cost effectiveness^21^, ecological validity and cultural needs are also important in selecting such tools.^22^ With these referential points, the Modified URLS in the current study is focusing on domains of receptive language i.e. attention, play, command following, understanding of gestures, morphology, syntax and semantics being similar to the domains of auditory comprehension subscale of PLS 5.^23^ For expert review analysis CVR and CVI for URLS items was calculated in compliance to the methods used in other studies for validation.^24^,^25^

A receptive vocabulary test in isiZulu language in preschool children was developed with a Cronbach alpha of 0.793 and the author emphasized on the significance of receptive language while stressing that it lays the foundation of expressive language development, unfolding literacy skills including reading and writing.^26^ Compared to this the Cronbach alpha of Urdu receptive language scale is even better and similar to other studies conducted for language assessments adaptations for Spanish, Russian, Turkish, isiZulu and protégées languages.^27^,^28^

### Recommendations:

Future study should be conducted on norm group across Pakistan for standardization, developing percentile ranks and norm scoring for URLS.

### Limitations:

Data of infants was in some cases collected through observation by parents this age group don’t respond every time, data from few experts was collected online. The scope of this research was limited to receptive language domain.

## CONCLUSIONS

The modified 57 items URLS has high validity and internal consistency with protocol of administration and it is linguistically appropriate and suitable for the application on larger scale with children of different cultural backgrounds in Pakistan.

### Authors’ Contribution:

**GAB and NM:** Did the manuscript writing.

**NM:** Conceived, designed and did the editing of manuscript.

**GS:** Did the critical revision, literature review and responsible for integrity of the research.
